# Cytokine-Mediated Crosstalk Between Keratinocytes and T Cells in Atopic Dermatitis

**DOI:** 10.3389/fimmu.2022.801579

**Published:** 2022-04-07

**Authors:** Mélanie Humeau, Katia Boniface, Charles Bodet

**Affiliations:** ^1^ Laboratoire Inflammation Tissus Epithéliaux et Cytokines LITEC UR 15560, Université de Poitiers, Poitiers, France; ^2^ ImmunoConcEpT, Centre National de la Recherche Scientifique, Unité Mixte de Recherche 5164, University of Bordeaux, Bordeaux, France

**Keywords:** cytokines, T cells, keratinocytes, atopic dermatitis (AD), *staphylococcus aureus*

## Abstract

Atopic dermatitis (AD) is a chronic inflammatory skin disease characterized by barrier dysfunction, dysregulated immune response, and dysbiosis with increased *Staphylococcus aureus* colonization. Infiltration of various T helper cell subsets into lesional skin and subsequent cytokine release are a hallmark of AD. Release of cytokines by both T cells and keratinocytes plays a key role in skin inflammation and drives many AD features. This review aims to discuss cytokine-mediated crosstalk between T cells and keratinocytes in AD pathogenesis and the potential impact of virulence factors produced by *Staphylococcus aureus* on these interactions.

## Introduction

Atopic dermatitis (AD) is one of the most common chronic inflammatory skin diseases, affecting approximately 15% to 25% of children and 3% to 10% of adults worldwide (1, 2). Lesions generally appear during childhood and/or during the third decade of life. AD is characterized by eczematous skin lesions, xerosis, erythematous scaling plaques and intense pruritus during flare-ups (1). The pathogenesis of AD is complex and involves multiple combinatorial factors, including genetic risk factors associated with immune dysregulation, mainly related to type 2 inflammation, and environmental stimuli, including allergens, stress, and microbial dysbiosis with dominant *Staphylococcus aureus* colonization (3, 4). AD is often associated with elevated serum concentrations of immunoglobulin E and a personal or family history of type I hypersensitivity, with an increased risk of developing food allergy, allergic rhinitis or asthma, called the atopic march, together with other comorbidities such as obesity, cardiovascular diseases, and cancers (5, 6). One of the classical features of AD is a strong type 2 immune response that leads to skin barrier dysfunction, such as inhibition of epidermal differentiation and increased skin permeability (3). Inflammatory cell infiltration found in lesional AD skin is characterized by the presence of type 2 innate lymphoid cells (ILC2) and various T cell subpopulations producing cytokines driving a T helper (Th) type 2-predominant inflammation (7, 8). Keratinocytes, the predominant cell type of the epidermis, are also key players in the AD pro-inflammatory environment (9). These cells constitute a major source of cytokines, including interleukin (IL)-25, IL-33, and Thymic stromal lymphopoietin (TSLP), chemokines, and antimicrobial peptides that orchestrate infiltration of T cells as well as innate immune cells (such as ILCs and mast cells) in lesional skin (9). Moreover, *S. aureus*-associated dysbiosis contributes to immune dysregulation and barrier dysfunction. This review aims to focus on the role of cytokines in the interplay between skin T cells and keratinocytes in AD physiopathology and to discuss the potential impact of virulence factors produced by *S. aureus* on this crosstalk.

## T Cells in AD

Skin infiltration of various CD4+ Th and CD8+ cytotoxic T cells (Tc) subsets is strongly implicated in AD pathogenesis (8, 10–12). Atopic skin is characterized by a predominant type 2 immune response, with Th2/Tc2 cells being involved in many features of AD. These cells are the main source responsible for increased levels of the type 2 cytokines IL-4, IL-5, IL-13, and IL-31 (12, 13), and infiltration of ILC2 in lesional AD skin also contributes to elevation of IL-13 and IL-5 levels (14). In addition, numerous studies have revealed the heterogeneity of T cell sub-populations infiltrating AD skin lesions, with the presence of Th2/Tc2, Th22/Tc22, Th17, and Th1 cells (10, 12, 15–18). Compared to healthy skin, non-lesional AD skin displays increased T cell infiltration and expression of Th2, Th22, and Th1-related cytokines and shares features with lesional AD skin, suggesting a global dysregulation of the T cell immune response in the skin of AD patients (12, 19–21). Similarly, blood of AD subjects harbors heterogeneous T cell subsets with an increased frequency of Th2/Tc2 and Th22/Tc22 cells compared to healthy donors (22–24).

This excessive effector response could be the result of a tolerance breach with a dysbalance of effector versus regulatory T cells (Tregs) and/or Tregs dysfunction in AD patients. Tregs are critical in the development and maintenance of self-tolerance mediated by immunosuppressive cytokine (25). Many studies have highlighted a dysregulated proportion of Tregs in lesional skin and peripheral blood of AD patients, while upward or downward variations have been reported depending on patient cohorts (22, 25–31). In addition, the deficiency of Tregs function can contribute to the development of AD. Indeed, Tregs dysfunction may be related to their propensity to differentiate into effector-like T cells under the influence of cytokines overexpressed in AD patients, leading to the expansion of pathogenic Tregs such as circulating Th2-like Tregs (31–33).

Importantly, persistence of identical T-cell clones in lesional and non-lesional AD skin suggests the involvement of resident memory T (T_RM_) cells, which are involved not only in disease progression and flares, but also during relapse of the disease (34), as previously shown in other chronic inflammatory dermatoses, such as psoriasis and vitiligo (35–37). Indeed, mouse models of allergic contact dermatitis have highlighted the presence of CD4+ and CD8+ T_RM_ cells in lesions, expressing inhibitory checkpoint receptors to control their reactivation in order to avoid the development of severe pathology (38, 39). So far, few studies have focused on the phenotype and function of T_RM_ cells in human AD skin and revealed their ability to produce multiple cytokines, including IL-4, IL-17A, IL-22 and IFN-γ (10, 40, 41). Interestingly, a recent study identified an enriched CXCR4+ T_RM_ natural killer T cell population in AD skin that may contribute to AD (42). Although these studies highlight the possible role of T_RM_ cells in AD, a more precise characterization of T_RM_ cell phenotype in AD lesions appears relevant to better decipher their function in disease development.

Recent development of single-cell RNA technology brought new insight of the characterization of T cells in AD skin (10, 18, 43, 44). The lesional AD samples were characterized by expansion of T_RM_, Th2/Tc2, and Th22 cells. Lesional T cells showed strong expression of type 2 (IL-13), type 17 (IL-26) and partly also of Th22 (IL-22) cytokines in activated T cells, proliferating T cells, and NK T cells (10, 18). Elevated expression of IL-26 was found to be associated with low levels of IL-17A in adult AD lesions, suggesting that Th17 cells are functionally deviant during disease progression. Pathway enrichment analysis revealed enrichment of the immune response, antigen processing and presentation, and regulation of apoptosis in lesional AD T cells. In healed AD lesion of adults, it was suggested that a Th1-skewed immune profile is involved in the clinical remission (44). Interestingly, identification of specific populations of disease-linked immune cells maintaining an inflammatory phenotype in resolved AD, including Th2 and Tc2 cells, suggest that these cells can be crucial for disease recurrence (43).

## Cytokines: Key Players in the Crosstalk Between T Cells and Keratinocytes in AD Skin

Through the release of cytokines in response to environmental stimuli such as microbial antigens and allergens, keratinocytes contribute to ILC2 and Th2 cell activation and hyperreactivity of AD skin. The resulting inflammatory environment drives many AD features such as barrier dysfunction, itch, defect in antimicrobial peptide production and infiltration of both innate and adaptive immune cells. Cytokine-mediated crosstalk between T cells and keratinocytes thereby plays a key role in AD onset and progression. Nevertheless, AD is a heterogenous disorder with differences in the immune phenotypes depending on the racial group and age that may impact the predominance of T cell subsets (45, 46).

### Impact on Barrier Dysfunction and Pruritus

**Figure 1:**
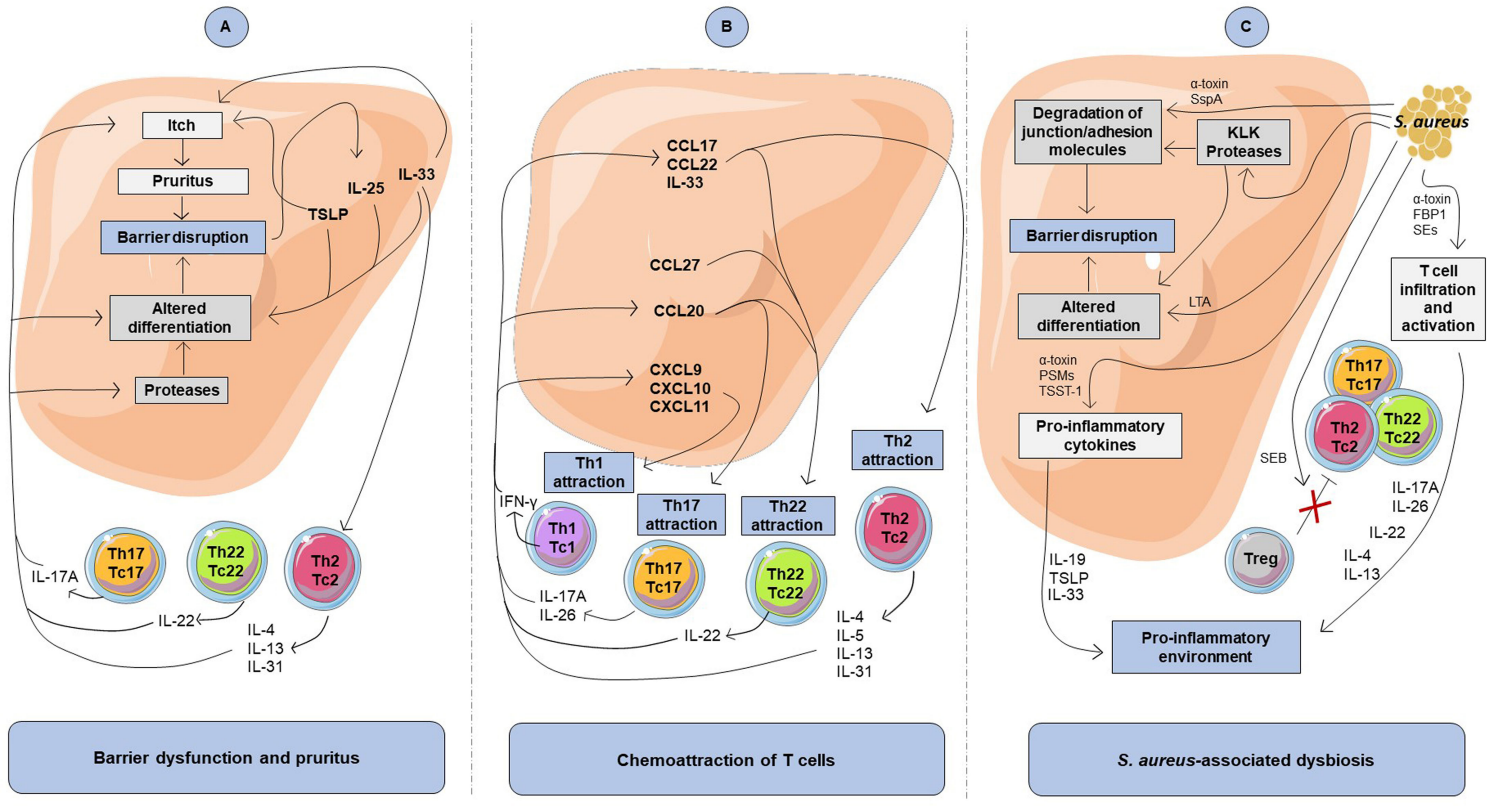
Impact of cytokine-mediated crosstalk between keratinocytes and T cells on atopic dermatitis features. **(A)** Involvement of the cytokine-mediated crosstalk in barrier dysfunction and pruritus. Th2, Th17, and Th22 cytokines inhibit differentiation markers and favor itch, leading to barrier disruption, which in return induces secretion of pro-inflammatory cytokine including TSLP, IL-33, and IL-25 by keratinocytes. **(B)** Involvement of the cytokine-mediated crosstalk in chemoattraction of T cells. The keratinocyte-derived inflammatory mediators include chemokines that lead to chemoattraction of T cells. Attraction of T cells and Th2 response promote a pro-inflammatory cytokine production loop in keratinocytes. **(C)** Impact of *S. aureus* on keratinocytes and T cells in atopic dermatitis. Virulence factors produced by *S. aureus* can promote degradation of junctional adhesion molecules and alteration of keratinocyte differentiation that contribute to atopic skin barrier disruption. *S. aureus* can also favor a pro-inflammatory environment through Th2 cell infiltration into the skin, pro-inflammatory cytokine release by keratinocytes and T cells as well as by inhibiting Treg immunosuppressive activity. FBP1, fibronectin-binding protein-1; LTA, lipoteichoic acid; PSMs, phenol soluble modulins; SEs, staphylococcal enterotoxins; SspA, serine protease A; TSST-1, toxic shock syndrome toxin-1.

Proteins expressed during the keratinocyte differentiation process leading to cornified envelope formation are responsible for the stratum corneum barrier function. Epidermal barrier dysfunction in lesional AD skin is partly related to a decreased filaggrin level and tight junction abnormalities. Numerous upregulated cytokines in AD lesions, including IL-4, IL-13, IL-31, IL-22, IL-17A, and oncostatin M, are known to alter barrier function through the inhibition of epidermal barrier protein synthesis (corneodesmosin, filaggrin, involucrin, loricrin, keratin-10), tight junctions (desmocollin, ZO-1, claudin-1, and -4), and/or lipids (fatty acid elongases ELOVL3 and ELOVL6, glucocerebrosidase, EO ceramides) (47–58). Interestingly, IL-24, whose expression is increased in Th2 cytokine-stimulated keratinocytes and epidermis from AD patients, has been suggested as a pivotal mediator for inhibition of keratinocyte differentiation (59–61). Moreover, IL-4 and IL-13 increase the expression in keratinocytes of kallikrein (KLK)5 and KLK7, proteins known to be upregulated in the stratum corneum of AD patients (51, 62). KLKs are key proteases involved in degradation of intercellular adhesion molecules, leading to desquamation, inhibition of barrier integrity and induction of pro-inflammatory cytokine production by keratinocytes, including TSLP (63). In addition, barrier disruption facilitates the entry of microbial products and allergens acting as danger signals that stimulate the release of AD-typical epidermal alarmins (TSLP, IL-25, and IL-33) from keratinocytes (64, 65). These alarmins activate immune cells that populate the skin, including T cells and ILCs, and are major contributors to allergic and type 2 immune response. In addition, they decrease filaggrin, claudin-1 and involucrin expression in keratinocytes (66–70). Through cytokine secretion, both T cells and keratinocytes thereby contribute to the disruption of skin barrier integrity (**Figure 1A** and **Table 1**).

**Table 1:** Role of cytokines produced by T cells and keratinocytes in AD features.

Cytokines	Major source cells	Target cells	Biological effects	AD features	References
**CCL17 CCL22**	Keratinocytes	T cells	Th2 and Th22 cells homing	Pro-inflammatory response	(71)
**CCL20**	Keratinocytes	T cells	Th22 and Th17 cells homing	Pro-inflammatory response	(72)
**CCL27**	Keratinocytes	T cells	Th22 cells homing	Pro-inflammatory response	(73, 74)
**CXCL9 CXCL10 CXCL11**	Keratinocytes	T cells	Th1 cells homing	Pro-inflammatory response	(75, 76)
**IL-4**	Th2/Tc2	Keratinocytes	IL-19 and IL-33 induction	Pro-inflammatory response	(77–81)
		T cells	TSLPR induction		
**IL-4**	Th2/Tc2	Keratinocytes	Protease and TSLP induction	Barrier disruption, pruritus, pro-inflammatory AMP and responses	(47, 50, 51, 53, 54, 56, 57, 82, 83)
**IL-13**			Differentiation marker, tight junction, lipid, hBD-2/3 and LL-37 inhibition		
**IL-17A**	Th17/Tc17	Keratinocytes	Differentiation marker inhibition	Barrier disruption, pro-inflammatory and AMP responses	(53, 58, 72, 80, 81, 84–86)
			IL-19, CCL20 and hBD-2/3 induction		
		T cells	Increases IL-4-producing cells		
**IL-18**	Keratinocytes	T cells	IL-4 and IL-13 induction	Pro-inflammatory response	(87)
**IL-19**	Keratinocytes	T cells	Th2 response promotion	Pro-inflammatory response	(88, 89)
	T cells				
**IL-22**	Th22/Tc22	Keratinocytes	Differentiation marker inhibition	Barrier disruption, pruritus, pro-inflammatory and AMP responses	(52, 53, 86, 90, 91)
			IL-33, TSLP, hBD-2/3 and S100A7/8/9, induction		
**IL-24**	Th2	Keratinocytes	Filaggrin and loricrin inhibition	Barrier disruption	(59–61)
	Keratinocytes				
**IL-25**	Th2/Tc2 Keratinocytes	Keratinocytes	Filaggrin inhibition	Barrier disruption, pro-inflammatory response	(66, 67, 92–94)
		T cells	Promotes Th2 phenotype with IL-4, IL-13, IL-5 secretion and IL-25R expression		
			Decreases Th17 cell frequency		
**IL-26**	Th17/Tc17	Keratinocytes	CCL20, IL-33 and hBD-2 induction	Pro-inflammatory and AMP responses	(95)
**IL-31**	Th2/Tc2	Keratinocytes	Differentiation marker and lipid inhibition	Barrier disruption, pruritus, pro-inflammatory and AMP responses	(49, 50, 55, 96–98)
			TSLP, CCL17, CCL22, hBD-2/3 and S100A7/8/9 induction		
**IL-33**	Keratinocytes	Keratinocytes	Differentiation marker inhibition	Barrier disruption, pruritus, pro-inflammatory response	(69, 70, 96, 99, 100)
		Th2 cells	Favors IL-31 secretion and direct chemoattraction		
**TSLP**	Keratinocytes	Keratinocytes	Filaggrin, S100A7 and hBD-2 inhibition	Barrier disruption, pruritus, pro-inflammatory and AMP responses	(68, 78, 99, 101–103)
		Th2 cells	Increases Th2 cells proportion		
			IL-4, IL-5 and IL-13 induction		

Cytokines released by Th2 and Th22 cells, in particular IL-31, IL-4, IL-13 and IL-22, can aggravate barrier alteration through itch-induced scratching. Th2 cells are the major contributor of pruritus *via* their production of IL-31, known as a pruritogen factor inducing nerve fiber elongation and branching (96). Moreover, IL-4, IL-13, IL-31, IL-33, human β-defensins (hBD)-2 and TSLP are known to directly activate sensory neurons, which cause itching (82, 97, 99, 101, 104). In addition, activation of sensory neurons by IL-4 and IL-13 through IL-4Rα signaling intensifies itch responses to other pruritogens such as IL-31 and histamine (97). Otherwise, IL-22 has been shown to cause chronic pruritus and induces keratinocyte expression of TSLP and IL-33 (90), which in turn contributes to itch response. Consequently, pruritogenic cytokines produced by T cells and keratinocytes promote atopic itch and promote barrier disruption and entry of allergens and dysbiotic bacteria, which may increase skin inflammation (**Figure 1A** and **Table 1**).

### Impact on Chemoattraction and Polarization of T Cells

Keratinocytes can amplify the attraction and production of cytokines by various subsets of T cells through their release of chemokines, including CCL17, CCL22, CCL20, CCL27, CXCL9, CXCL10 and CXCL11 (73, 75, 105, 106), whose expression is increased in AD skin (15, 19, 107–112). These chemokines influence the skin T cell environment, with recruitment of circulating effector memory Th2, Th22, Th1, and Th17 cells, highlighting the complexity of AD pathogenesis (**Figure 1B**). In addition, IL-33 secreted by keratinocytes from AD patients exerts a direct chemoattractant activity on Th2 cells (100). Once attracted, T cell subsets will further amplify the inflammatory response through the promotion of chemokines secretion by skin cells, including keratinocytes (50, 72, 75, 77, 84, 95, 98, 113).

In addition to T cell chemoattraction, keratinocyte-derived cytokines may favor exacerbation of a skewed T cell inflammatory response (**Figure 1B** and **Table 1**). Indeed, increased secretion of TSLP, IL-33 and IL-25 by epidermal cells can promote secretion of cytokines by Th2 cells (114–116). IL-33 potentiates IL-31 secretion by Th2 cells (96), while TSLP and IL-25 directly promote Th2 cell polarization as well as IL-4, IL-13, and IL-5 secretion (78, 92, 93, 102) which exacerbates barrier disruption through potentiation of the type 2 inflammatory response. In addition, IL-25, produced by both Th2 cells and keratinocytes (117), increases IL-25R receptor expression on Th2 cells, further amplifying IL-25-induced Th2 cytokine secretion (92, 93). Recently, the prominent role of IL-25 was highlighted in a mouse model of AD, which showed that IL-25 is essential for IL-13 production and contributes to epidermal thickening, CD4+ T cell infiltration and the expression of the Th2 cell–attracting chemokines CCL17 and CCL22 (115).

In turn, cytokines released by Th2 and Th22 cells may increase type 2 cytokine secretion by keratinocytes (**Table 1**). Indeed, IL-4, IL-13, IL-22 and IL-31 induce TSLP, IL-33, CCL17 and CCL22 expression by keratinocytes, both in 2D cultures (77) and 3D models of reconstructed human epidermis (50, 90, 98), a phenomenon that may reinforce skin homing of Th2 and Th22 cells. Moreover, IL-4 increases the expression of the receptor TSLPR on CD4+ T cells, which may promote IL-4 induction by TSLP (78, 79), suggesting a positive regulatory loop between Th2 cells and TSLP. Therefore, the cytokine crosstalk between keratinocytes and T cells reinforces AD as a Th2- and Th22-polarized disease.

Furthermore, IL-18 production by keratinocytes is increased in the epidermis of AD patients (118) and contributes to Th1/Th2 balance regulation that might favor AD physiopathology. IL-18 induces IL-4 and IL-13 production by T cells (87). Moreover, addition of IL-18 on IFN-γ-treated keratinocytes further increases CXCL10 secretion (119). Predominance of Th2 cytokines in AD lesions may regulate Th17 response, IL-25 being known to decrease Th17 cell through inhibition of Th17-inducing cytokine release (94). On the other hand, Th17 cells may also favor a Th2 environment. IL-17A induces IL-19 secretion by keratinocytes (80, 81), a cytokine stimulating the production of Th2 cytokines (88, 120). Moreover, in a mouse model of AD, IL-17A has been shown to mediate Th2 immune response by inducing TSLP and CCL17 expression and IL-4-producing cells (85). In addition, the Th17-derived cytokine IL-26 increases in lesional AD skin and induces CCL20 and IL-33 expression by keratinocytes, suggesting a role of IL-26 for bridging between Th17 and Th2 responses in AD (95). These data suggest that IL-18, IL-19 and IL-26 could be important cytokines of the keratinocyte-T cell crosstalk in AD, which may require further exploration.

### Impact on Antimicrobial Peptide Production

The modest increase of antimicrobial peptides (AMPs), such as hBD-2, hBD-3, or LL-37, observed in AD skin as compared to psoriasis lesions, results mainly from the type 2 cytokine microenvironment which limits AMP production by keratinocytes (121, 122). On the one hand, IL-4, IL-13, IL-33, and TSLP inhibit hBD-2, hBD-3, and LL-37 expression (83, 103, 123). On the other hand, other cytokines such as IL-1β, IL-17A, IL-26, IL-22, and IL-31 can stimulate hBD-2, hBD-3, and S100A production by keratinocytes (86, 91, 95, 96, 124). Furthermore, AMPs can initiate negative feedback on their production through Th2 cytokine induction. For example, hBD-2 and hBD-3 induce IL-13 and IL-4 and inhibit IL-17A secretion by T cells (86). Impaired expression of AMPs, key components of keratinocyte defenses against microorganisms, can lead to perturbed innate immunity and favor susceptibility to skin infections and dysbiosis in AD patients.

## Role of the Cutaneous Dysbiosis on T Cell and Keratinocyte Crosstalk

AD patients are characterized by skin dysbiosis, with dramatically reduced diversity of cutaneous microbiota and overabundance of staphylococci (mainly *S. aureus* and *S. epidermidis*) on lesional AD skin, correlated to disease severity (125–128). A meta-analysis reported that *S. aureus* colonizes 70% of lesional AD skin and 39% of non-lesional AD skin (129). During lesion recovery or treatment of AD flares, skin bacterial diversity is improved and *S. aureus* proportion is decreased (128, 130). Some AD features, such as filaggrin deficiency and altered AMP production, associated with elevated skin pH, may favor *S. aureus* colonization of lesional skin (131–135). *S. aureus* can counteract cutaneous antibacterial defense through various AMP resistance mechanisms (136–138) and potentiate skin inflammatory response and barrier dysfunction through secretion of various virulence factors, including superantigens, enterotoxins (SEs), fibronectin-binding protein-1 (FBP1), phenol-soluble modulins (PSMs), α-toxin, and proteases (139).

Firstly, *S. aureus* and *S. epidermidis* can worsen AD skin barrier disruption by acting directly on keratinocytes. *S. aureus*-derived compounds, such as lipoteichoic acid (LTA), have been shown to inhibit terminal differentiation of keratinocytes (140, 141). In addition, ɑ-toxin secreted from *S. aureus* induces skin barrier disruption and cell death on filaggrin-deficient keratinocytes (142, 143), thereby contributing to AD exacerbation. In addition, *S. aureus* serine protease A (SspA) and *S. epidermidis* cysteine protease (Ecpa) cleave tight junction proteins and degrade adhesion molecules in the epidermis (125, 144). Finally, *S. aureus* enhances keratinocyte KLK proteolytic activity, leading to degradation of differentiation proteins such as filaggrin and desmoglein-1 (145). As a result, through various virulence mechanisms, staphylococci-associated dysbiosis promotes disruption of barrier integrity (**Figure 1C**).

In addition, *S. aureus* is thought to exacerbate skin inflammation in AD patients through the activity of cytolytic toxins and superantigens. On the one hand, it has been shown that many virulence factors of *S. aureus* promote keratinocyte pro-inflammatory response. *In vitro*, PSMs, α-toxin, and TSST-1 induce secretion of pro-inflammatory cytokines and chemokines from keratinocytes (143, 146–148). Interestingly, dysbiosis-driven intracellular IL-1α release from keratinocytes has been shown to trigger chronic skin inflammation in filaggrin-deficient mice (149). Concordantly, studies of epicutaneous *S. aureus* colonization on mice have revealed that PSMα peptide drives skin inflammation through production of IL-1α and IL-36α by keratinocytes, leading to subsequent IL-17A secretion by T cells (150–152). On the other hand, *S. aureus* superantigens and cytotoxins enhance cytokine secretion by various effector T cells (153–156). Moreover, staphylococcal superantigens have the ability to bind directly to the major histocompatibility complex class II from antigen-presenting cells and to the T cell receptor without antigen presentation (157), leading to non-specific T cell activation and abundant cytokine secretion (158). For example, the staphylococcal enterotoxins SEE and SEA induce IL-26 secretion by T cells (159), a cytokine promoting AD development (95). At sublytic concentrations, in the absence of antigen-presenting cells α-toxin also activates T cells by upregulating IFN-γ and IL-17A secretion by CD4+ T cells (160, 161).

Furthermore, some data have shown that *S. aureus*-associated dysbiosis can favor types 2 and 22 immune responses in AD skin. *S. aureus* components trigger the release of TSLP, IL-33, and IL-19 in keratinocytes (162–164), which could amplify cytokine secretion by Th2 cells. Moreover, cutaneous application of staphylococcal peptidoglycan or δ-toxin on mouse skin has highlighted the involvement of *S. aureus* in increasing cutaneous infiltration of CCR4+ cells associated with cytokine secretion leading to Th2-dominant inflammation (165, 166). Concordantly, in an epicutaneous sensitization mouse model, topical application of live *S. aureus* or SEB increase cutaneous accumulation of T cells and type 2 cytokine expression, thereby highlighting a mutually reinforcing role of allergic inflammation and *S. aureus* colonization in AD skin (167, 168). Moreover, repeated topical applications of *S. aureus* to mouse skin reproduce AD-like skin inflammation with T_RM_ cells accumulation and IL-4 and IL-17A increased expression (169). In filaggrin-deficient mice, *S. aureus* entry into the skin is associated with increased expression of inflammatory cytokines, including IL-17A, IL-22 and the type 2 cytokines TSLP, IL-13, and IL-4 (134). Furthermore, SEB, α-toxin, and FBP1 can induce T cell proliferation and production of IL-31, IL-4, IL-13, and IL-22 by T cells (170–172). To summarize, there is ample evidence that virulence factors of *S. aureus* may promote Th2, Th17 and Th22 cytokine secretion and influence the phenotypic profile of T cells in AD skin, thereby contributing to disease persistence (**Figure 1C**).

Finally, the intensity of the inflammatory response may be potentiated by *S. aureus* through several strategies designed to avoid Tregs immunosuppressive activity. Stimulation of Tregs with *S. aureus* secretome and SEB inhibits their suppressive functions on conventional T cell proliferation, suggesting that *S. aureus* favors T cell-dependent skin inflammation (29, 173, 174). In addition, reprogramming of Tregs toward a Th2-like phenotype after activation by *S. aureus* superantigen has been reported (175) and could contribute to the predominance of Th2 effector cells in AD.

## Conclusion

Notwithstanding the type 2 response predominance, the AD inflammatory environment is the result of cytokine release from various skin T cell subpopulations, including Th2/Tc2, Th22/Tc22, Th17/Tc17 and Th1/Tc1 subsets, associated with Tregs dysfunction. The crosstalk between T cells and keratinocytes is involved in barrier dysfunction and favors both attraction of T cells and persistence of T_RM_ cells in AD lesions, thereby promoting consistent release of cytokines. The dialogue between T cells and keratinocytes is mediated mostly *via* a complex dynamic network of cytokines and chemokines. This inflammatory environment resulting from the T cell-keratinocyte interactions is also involved in altered AMP production characteristic of AD, which may favor colonization and persistence of *S. aureus* on AD lesions. In return, secreted virulence factors by *S. aureus* act on barrier disruption and exert pro-inflammatory effects on both keratinocytes and T cells, impacting disease-driving mechanisms. Further investigations are needed to better understand the impact of this immune dialog and dysbiosis on the dynamics of skin inflammation in AD patients.

## Author Contributions

MH wrote the first draft. KB and CB contributed to the revision of the manuscript. All authors approved the final version of the manuscript.

## Conflict of Interest

The authors declare that the research was conducted in the absence of any commercial or financial relationships that could be construed as a potential conflict of interest.

## Publisher’s Note

All claims expressed in this article are solely those of the authors and do not necessarily represent those of their affiliated organizations, or those of the publisher, the editors and the reviewers. Any product that may be evaluated in this article, or claim that may be made by its manufacturer, is not guaranteed or endorsed by the publisher.

## Abbreviations

AD, atopic dermatitis; AMP, antimicrobial peptide; CCL, chemokine (C-C motif) ligand; FBP1, fibronectin-binding protein-1; hBD, human beta-defensin; IFN, interferon; IL, interleukin; KLK, kallikrein; LTA, lipoteichoic acid; PSM, phenol-soluble modulin; TARC/CCL17, thymus and activation regulated chemokine; MDC/CCL22, macrophage-derived chemokine; SspA, serine protease A; SE, staphylococcal enterotoxin; Tc, cytotoxic T cells; Th, T helper cells; Treg, regulatory T cells; T_RM_, resident memory T cells; TSLP, thymic stromal lymphopoietin.
